# Comparison of the physiological characteristics of transgenic insect-resistant cotton and conventional lines

**DOI:** 10.1038/srep08739

**Published:** 2015-03-04

**Authors:** Xiaogang Li, Changfeng Ding, Xingxiang Wang, Biao Liu

**Affiliations:** 1 Key Laboratory of Soil Environment and Pollution Remediation, Institute of Soil Science, Chinese Academy of Sciences, Nanjing 210008, China; 2 Nanjing Institute of Environmental Sciences, Ministry of Environmental Protection of China, Nanjing 210042, China

## Abstract

The introduction of transgenic insect-resistant cotton into agricultural ecosystems has raised concerns regarding its ecological effects. Many studies have been conducted to compare the differences in characteristics between transgenic cotton and conventional counterparts. However, few studies have focused on the different responses of transgenic cotton to stress conditions, especially to the challenges of pathogens. The aim of this work is to determine the extent of variation in physiological characteristics between transgenic insect-resistant cotton and the conventional counterpart infected by cotton soil-borne pathogens. The results showed that the difference in genetic backgrounds is the main factor responsible for the effects on biochemical characteristics of transgenic cotton when incubating with cotton *Fusarium oxysporum*. However, genetic modification had a significantly greater influence on the stomatal structure of transgenic cotton than the effects of cotton genotypes. Our results highlight that the differences in genetic background and/or genetic modifications may introduce variations in physiological characteristics and should be considered to explore the potential unexpected ecological effects of transgenic cotton.

Cotton is an economically important crop in the world. Transgenic insect-resistant cotton expressing the Cry1Aa/c and/or CpTI protein(s) has been planted for over a decade in China, a nation prominent in pioneering the use of this new technology^1^. It can effectively control the cotton bollworm (*Helicoverpa armigera*), thus protecting the ecological environment by the reduced application of chemical insecticides, and exhibited favorable socioeconomic benefits^2^,^3^. Nevertheless, the introduction of transgenic insect-resistant plants into agricultural ecosystems has raised a number of questions; one of the major concerns regarding transgenic insect-resistant cotton is the ecological effect on non-target organisms^4^,^5^,^6^.

It is crucial that risk assessment studies on the commercial use of transgenic insect-resistant crops consider the impacts on organisms inside and outside the soil. The effects of transgenic insect-resistant cotton on non-target pests, natural enemies and pollinators have been extensively assessed^7^,^8^,^9^. However, convincing negative effects on the growth, abundance and diversity of transgenic insect-resistant cotton have not been found^7^,^8^,^9^. Transgenic insect-resistant crops have the potential to influence soil-dwelling organisms and essential ecosystem functions in soil because they usually produce insecticidal Cry proteins throughout all parts of the plant^10^,^11^,^12^. In general, few or no toxic effects of Cry proteins on woodlice, collembolans, mites, earthworms, nematodes, protozoa, and microbial communities in soil have been reported^10^,^11^,^12^,^13^.

Nevertheless, some researchers have raised concerns that the transformation process could result in various unintended effects, which are unrelated to the nature of the specific transgene^14^,^15^,^16^. Although most event-specific effects are routinely eliminated during the early screening stages^17^, there are some reports of apparently normal transgenic plants exhibiting aberrant behavioral or biochemical characteristics upon further analysis^16^,^18^. For example, higher lignin levels and composition were found in stems of the MON810 Novelis T and Valmont T varieties than their respective near-isogenic lines^19^, and unforeseen metabolic variations involving the primary nitrogen pathway were observed when comparing La73-*Bt* (MON810) and La73 (non-transgenic)^20^. Several studies have also reported potentially unintended effects of transgenic plants exposed to a range of field conditions. Examples of these unexpected traits included lower yields^21^, an enhanced susceptibility to pathogens^22^, and altered insect resistance as a consequence of non-target changes in the secondary metabolism^23^.

*Fusarium* wilt, a vascular disease caused by soil-borne *Fusarium oxysporum* f. sp. *vasinfectum*, is a major constraint to cotton production not only in China but also worldwide^24^. Recently, the resistant attenuation of transgenic insect-resistant cotton to certain diseases, especially to *Fusarium* wilt, has been widely reported in China^25^,^26^. For example, Zhu *et al*. (2005) evaluated the disease resistance of 35 transgenic cotton varieties and found that the resistances of transgenic insect-resistant cotton were inferior to conventional cotton against both *Fusarium* wilt and *Verticillium* wilt^27^. Therefore, the unintended effects in the physiological resistance of transgenic insect-resistant cotton to certain pathogens should be explored.

The main objective of this study was to investigate the extent of physiological changes in the two transgenic insect-resistant cotton varieties when compared to conventional counterparts as well as to elucidate the causes for these unintended changes.

## Results

### Changes in antioxidant enzyme activities

**Figure 1:**
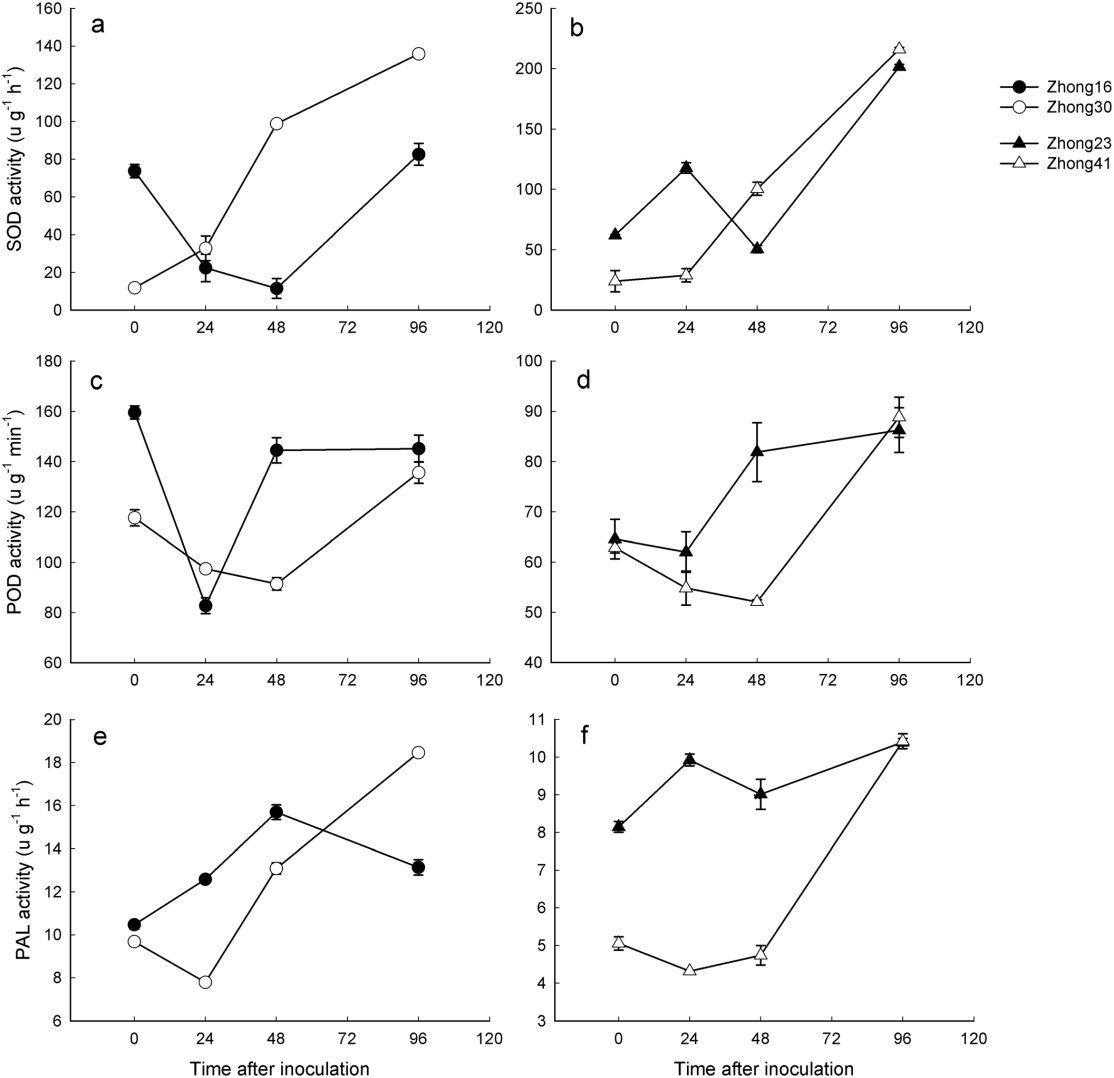
The variation in antioxidant enzyme (POD, SOD, PAL) activities in transgenic insect-resistant cotton lines, Zhong-30 and Zhong-41, and respective conventional counterparts, Zhong-16 and Zhong-23, when inoculated with *F. oxysporum*.

The repeated measures procedure of the generalized linear model showed that “genotype” and “pathogen infection” had significant effects on the activities of SOD, POD, whereas “transgenic” did not (Table 1). “Genotype”, “transgenic” and “pathogen infection” all had significant effects on the activities of PAL (Table 1). The results also indicated greater differences in these antioxidant enzymes activities between the two pairs of genotypes than between transgenic lines and their counterparts (Table 1). The comparative analysis with the respective conventional counterparts revealed that before pathogen inoculation, the SOD activities of transgenic lines were significantly lower (Fig. 1a, b) and the POD activities were significantly higher. However, the SOD activities were significantly lower at 48 h of pathogen infection (Fig. 1a, b, c, d) and the PAL activities were significantly lower across the most of the sampling period (Fig. 1e, f).

### Changes in soluble sugar and protein contents

**Figure 2:**
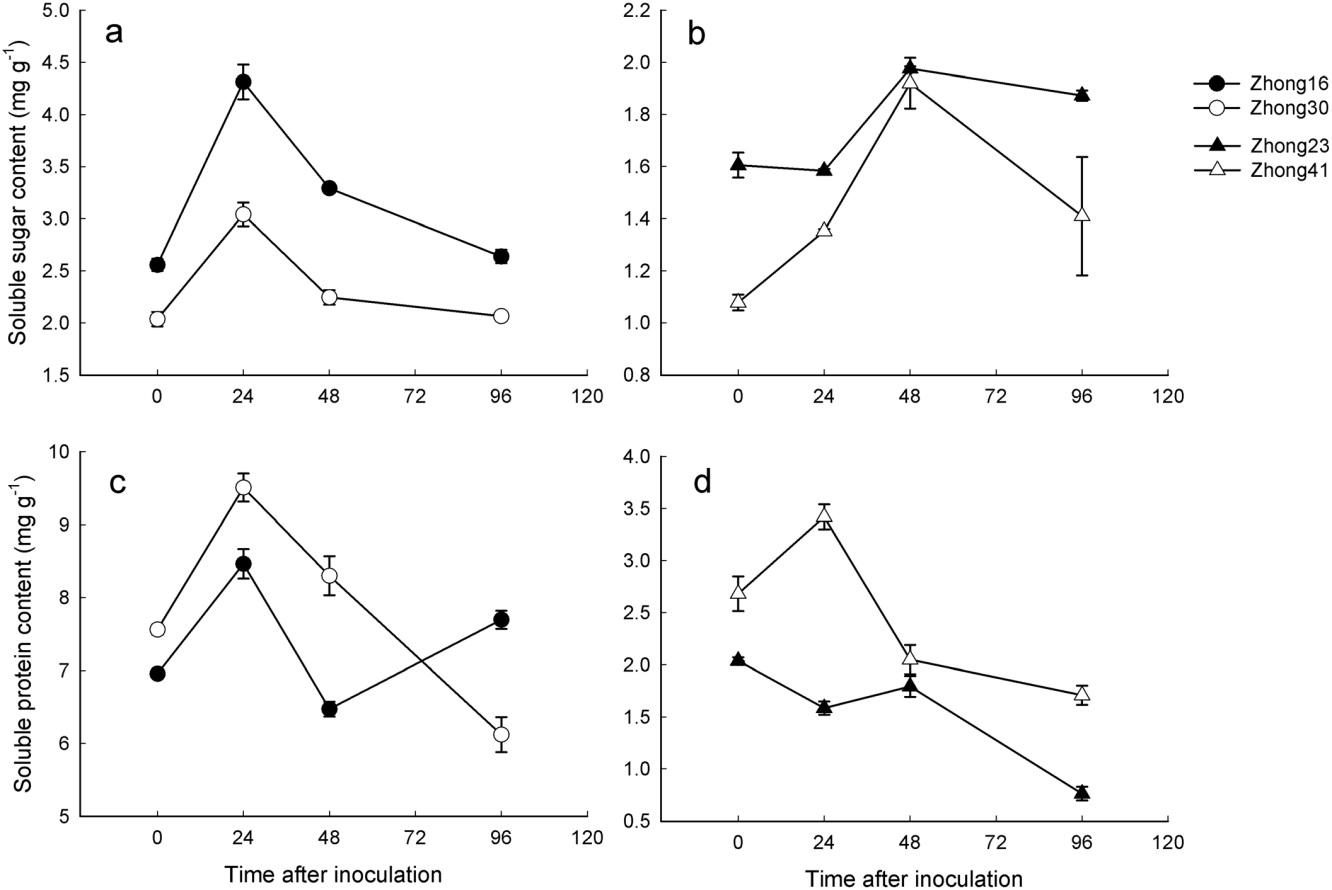
The variation of soluble sugar and protein contents in transgenic insect-resistant cotton lines, Zhong-30 and Zhong-41, and respective conventional counterparts, Zhong-16 and Zhong-23, when inoculated with *F. oxysporum*.

As shown in Table 1, “genotype” and “pathogen infection” had significant effects on the protein content, whereas “transgenic” did not. “Pathogen infection” had significant effects on the sugar content, whereas “genotype” and “transgenic” did not. Greater differences in these biochemical characteristics were found between the two-pair genotypes than between transgenic lines and their counterparts (Table 1). The comparative analysis with the respective conventional counterparts revealed that the soluble sugar contents of transgenic lines were significantly lower, yet the protein contents were significantly higher across most of the sampling period (Fig. 2a, b, c, d).

### Determination of leaf stomata

**Figure 3:**
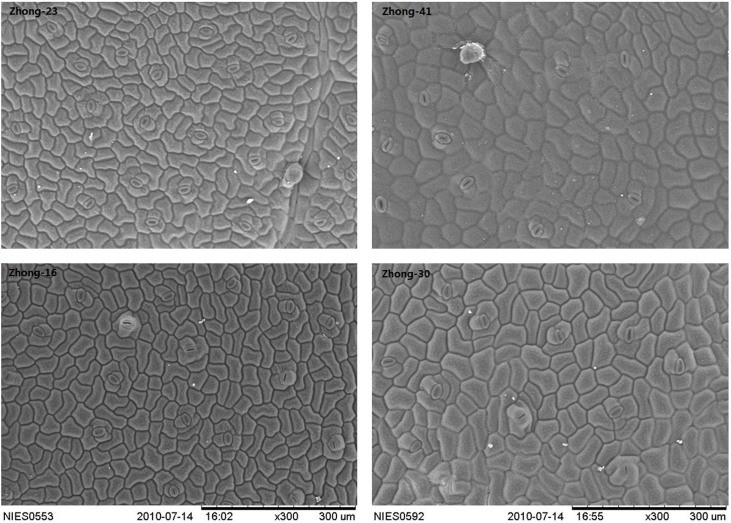
The shape and density of leaf stomata in transgenic insect-resistant cotton lines, Zhong-30 and Zhong-41, and their respective conventional counterparts, Zhong-16 and Zhong-23, at 300× magnification using a scanning electron microscope.

As determined by the two-way ANOVA, “transgenic” had significant effects on the transverse and longitudinal diameters of leaf stomata, whereas “genotype” did not (Table 2). Both “genotype” and “transgenic” had significant effects on the stomatal density (Table 2). The results also indicated greater differences in these stomatal characteristics between transgenic lines and their counterparts than between the two-pair genotypes (Table 2). As shown in Fig. 3, there were no significant variations in the shape of the leaf stomata between transgenic lines and their counterparts. The comparative analysis with their respective conventional counterparts revealed that the transverse and longitudinal diameters of leaf stomata in transgenic lines were significantly larger and the stomatal densities were significantly lower (overall, *P* < 0.05) (Table 3).

## Discussion

Many studies on the assessment of the unintended effects of transgenic crops have provided details of transcriptomic, proteomic and metabolomic differences between conventional and transgenic plants, including different species and traits^14^,^15^,^16^,^17^. However, studies on these pair-wise differences need to be examined in a wider context of natural variation^28^. To our knowledge, no data are available on the physiological responses of transgenic insect-resistant cotton facing the challenges of cotton pathogens. Therefore, in this study, we conducted preliminary studies on changes in the physiological characteristics of the two transgenic insect-resistant cotton varieties and their conventional counterparts.

Plants defend themselves against pathogen challenges by the activation of defense response pathways^29^. The known defense genes mainly encode pathogenesis-related proteins and various biosynthetic and antioxidant enzymes such as PAL, SOD, POD, etc.^30^,^31^,^32^. Our data revealed that genotypes had a stronger overall effect on the PAL, SOD and POD activities of the four cotton lines than the genetically modified variations. Comparative analyses assessing the proteome diversity of a range of non-transgenic potato germplasm and eight transgenic lines of potato demonstrated considerably fewer differences between the transgenic and non-transgenic lines of the same genetic background than between different non-transgenic cultivars^33^,^34^. Our results are consistent with these previous publications, suggesting that genetic background is the primary cause for the changes in biological enzyme activities of transgenic cotton^35^,^36^.

Plant stress responses are associated with a wide array of mechanisms involving increased demands for energy and its redistribution. Our analyses of the carbohydrate and protein metabolic profiling data indicated that cotton stress responses to pathogen inoculation can mask differences between samples from different genotypes or different transformations. However, the comparative analysis using the respective conventional counterparts revealed a significantly lower soluble sugar and higher protein content in transgenic lines across most of the sampling period (Fig. 2). The glycolysis pathway and protein synthesis are both competitive pathways, as phenylalanine produced by the shikimate pathway was an intermediate of protein metabolism. Therefore, the higher protein content of transgenic cottons infected by pathogens might inhibit the shikimate pathway, thereby affecting the synthesis of phenylalanine^37^. Indeed, the PAL activities in transgenic cotton lines were significantly lower across most of the sampling period when comparatively analyzed with their respective conventional counterparts (Fig. 1e, f).

It is noting that before *F. oxysporum* incubation some biochemical characteristics among cotton cultivars were significant differences, such as lower SOD, PAL activities and soluble sugar content, and higher protein content in transgenic cottons than their respective conventional counterparts, which was in part agree with the results of Chen *et al*. (2005) and Xu *et al*. (2011) who found *Bt* cultivars had lower oxido-reductase activities and more intense leaf nitrogen metabolism than non-transgenic cultivars^38^,^39^. Distinct variations in these biochemical characteristics of cottons after *F. oxysporum* incubation were determined, implying that *F. oxysporum* actually infected cotton plants when *in vitro* inoculating to roots in our experimental system, because rapid induction and high levels of defense gene expression are necessary for plants to defend against pathogens^31^,^32^. As the first enzyme in the phenylpropanoid pathway, PAL has important functions in plants following pathogen attack^40^. In active oxygen-scavenging systems, superoxide radicals generated in plants are converted to H_2_O_2_ by the action of SOD. POD is one of the most important enzymes for the elimination of reactive oxygen species (ROS) and catalyzes the oxidoreduction of various substrates using hydrogen peroxide^31^,^40^. Thus, transgenic cottons had lower values of PAL and POD, but higher SOD and protein at most time of pathogen inoculation than the respective conventional counterparts, indicating that the antioxidant enzymes appeared to eliminate ROS less efficiently in transgenic cottons, and as a result, oxidative damage is more. Therefore, our results suggest that the changes in these biochemical characteristics could be one of the causes for the declined resistance of transgenic lines to cotton pathogens including *F. oxysporum* in field investigations^25^,^26^,^27^.

Within the context of transgenic crops, the relevance of unintended effects is mainly related to their implications regarding agronomic performance^14^,^17^. There are examples showing that genetic modification may generate non-desirable phenotypic alterations as a consequence of pleiotropic changes in plant growth and development, compromising the preservation of the identity of the transformed genotype^28^,^41^,^42^. Our study found that genetic modification had a stronger overall effect on the stomatal sizes and stomatal density of cotton leaves than the different genotypes. However, a previous report of our study revealed that there were no significant differences in stomatal length and stomatal density between transgenic *Bt* rice and its counterpart^43^. Therefore, the different results in the different crop types suggest the specific event could occur in the transgenic breeding process. It was interesting that the density of trichomes covering the leaves of transgenic *Bt* cotton was significantly lower than that of its counterpart^41^. Some research has indicated that transgenic cotton cultivars had excessive vegetative growth, such as increased plant height^44^, higher biomass^45^ and nitrogen metabolism^38^. Therefore, the greater stomatal pore sizes of transgenic cotton determined in the study implied that transgenic cotton could take in more CO_2_, and then accelerate its growth. The ecological implication of our findings, including the different responses of transgenic cotton to global climate change and elevated O_3_, need further investigations.

A comparative assessment should always consider the extent of natural variation and not simply compare transgenic lines and parental controls^36^,^46^. A recent report reviewed 44 studies on the effect of genetic modifications, comparing environmental and inter-variety variation in several crops and *Arabidopsis*^28^. The authors reported that the most pronounced differences were observed between different varieties obtained by conventional breeding, and transgenesis had the least impact^28^,^47^. The breeding programs of transgenic insect-resistant cotton in China were generally used to acquire transgenic germplasm by means of conventional transformations, i.e., *Agrobacterium tumefaciens*-mediated transformations and pollen-tube pathways, which were then backcrossed to the local major cotton cultivars. Therefore, unintended changes in the physiological characteristics in transgenic insect-resistant cotton could be caused by the transgene integration in the genome or the differences in genetic background^9^,^15^,^18^,^28^,^33^. In our study, the results of the generalized linear model demonstrated that genetic background and genetic transformation may have the potential to introduce unintended variations in the physiological characteristics of transgenic insect-resistant cotton.

In conclusion, the results obtained here show that, at least in the material analyzed in this study, the difference in genetic background is the main factor responsible for the effects on the biochemical characteristics of transgenic cotton when incubating with *F. oxysporum*. This suggests that the unintended variation of biochemical resistance between transgenic and non-transgenic comparable cotton fall into the generally acceptable range, naturally occurring in different cultivars or lines of the species. However, genetic modifications had significantly greater influences on the stomatal structure of transgenic cotton than the effects of cotton genotypes. With regard to this, it is important to note that transgenic cotton could face greater challenges to global climate change, such as elevated O_3_ and drought. However, these pronounced differences between the transgenic and non-transgenic lines of the same genetic background should be verified in further pair-wise studies, as well as in other developmental stages. Our results highlight the differences in the physiological characteristics in transgenic cotton that might depend on genetic background and/or genetic transformation. Both causes should be considered to investigate the potential unexpected effects of transgenic cotton.

## Methods

### Cotton lines

The transgenic *Cry1Ab/c* gene cotton line Zhong-30 was bred by the sexual hybridization between "transgenic germplasm cotton line 110" (male parent, containing *Cry1Ab/c* gene) and "conventional line Zhong-16" (female parent) and then backcrossed with parental line Zhong-16. The transgenic *Cry1Ac* plus *CpTI* genes cotton line Zhong-41 was bred using a pollen-tube pathway transformation of a construct containing *Cry1Ac* plus *CpTI* into parental line Zhong-23. The two transgenic lines modified for the two different genes were representative of transgenic cotton and were commercially grown at a large scale for many years in China. Transgenic lines Zhong-41 and Zhong-30 and their conventional counterparts Zhong-23 and Zhong-16, respectively, were obtained from the Cotton Research Institute (CRI) of the Chinese Academy of Agricultural Sciences, Anyang, China. The CRI was also the breeder of these cotton lines mentioned above.

### Pathogen

The highly virulent strain of *F*. *oxysporum* f. sp. *vasinfectum* (Atk.) Snyder and Hansen was used. This fungus is a race-7 strain that causes *Fusarium* wilt disease and is responsible for significant yield losses of cotton throughout the world, including China. This strain was also obtained from the CRI.

### Soil preparation and plant growth

This experiment used a sandy-textured soil with the following properties (on a dry mass basis): pH (soil: water ratio 1: 2.5) 6.1, organic *C* 19.2 g/kg, total *N* 1.3 g/kg, total *P* 0.5 g/kg, total *K* 9.1 g/kg, available *P* 0.1 mg/kg, available *K* 46.2 mg/kg and soil clay (<0.002 mm) 24.3%. Fresh soil was sieved to pass through a 3 mm sieve and kept in darkness before use. Aliquots (1 kg) of soil were uniformly mixed with 0.05 kg of organic fertilizer and moist heat sterilized at 121°C for 3 h and heat sterilized again to obtain sterile soil for cultivation. The cotton seeds were surface-delinted in concentrated sulfuric acid and then immersed in sterile distilled water for 8 h. The cotton seeds were planted in plastic pots containing the sterile soil and then transferred to a greenhouse (day: 25–30°C, night: 20–25°C). Each cotton line was planted in triplicate, and each repetition contained fifteen cotton seedlings.

### Inoculation of fungal pathogen and sample collection

For the preparation of the conidial suspensions, a block of the stock culture was activated on PDA medium for 3 d at 28°C in the dark and then transferred to a new PDA medium for 5 d at 28°C in the dark. The mycelium from 5 Petri dishes was scraped and mixed with 25 mL sterile deionized water, which was blended two times. The spore suspension was concentrated (3000 × g, 10 min) in a sterile centrifuge tube and adjusted to a final concentration of 1 × 10^7^ conidia/mL in sterile distilled water using a hemocytometer. At the 3-leaf stage, the roots of four cotton lines were partially cut using a sterile knife blade and each plant was inoculated with 5 mL of a 1 × 10^7^ conidia/mL spore suspension. Soil moisture was kept at approximately 80% during incubation to be conducive to pathogen infection^48^. The leaves of four cotton lines were collected at (0), 24 h, 48 h, and 96 h post-inoculation, and the leaves were surface-washed with sterile distilled water three times and frozen in liquid nitrogen.

### Antioxidant enzyme extraction and assay

The activity in leaves was measured by the procedures described by Garcia-Limones *et al*. (2002)^32^. Fresh leaves of each cotton line (0.20 g) were homogenized in 1.5 mL 50 mM phosphate buffer (pH 7.8) and centrifuged at 10,000 × g for 10 min. All steps of the extraction procedure were conducted at 1–4°C. Superoxide dismutase (SOD) activity was assayed by measuring the ability of the enzyme extract to inhibit the photochemical reduction of NBT. In brief, the supernatants of each cotton line (0.5 mL) were added into a mixture containing 25 mM phosphate buffer (pH 7.8), 13 mM methionine (Met), 75 mM nitrotetrazolium blue tetrazolium (NBT), 10 mM EDTA-Na_2_, and 2.0 mM riboflavin. The above mixtures of each cotton line were placed in a light incubator (light intensity: 54 mmol/m/s) at 25°C for 20 min and were measured with a Multimodel Plate Reader (Infinite 500, Tecan, Switzerland) at 560 nm. One unit of SOD activity equals the amount of the enzyme that inhibits the rate of NBT reduction by 50% under these conditions. Peroxidase (POD) activity was determined using the following methods: the supernatant (0.1 mL), same as above, was added into a mixture containing 50 mM phosphate buffer (pH 7.8), 50 mM guaiacol, and 10 mM H_2_O_2_ and measured with the Multimodel Plate Reader at 470 nm. One unit of enzyme activity was designed as the change in absorbance of 0.01 for 1 g fresh weight per minute.

Phenylalanine ammonia lyase (PAL) activity was assayed using the absorbance of the amount of t-cinnamic acid, a product formed by deamination of phenylalanine via biochemical catalysis. Briefly, 0.20 g of fresh leaf tissue was homogenized with chilled Tris-HCl (0.05 M, pH 8.8), supplemented with β-mercaptoethanol (0.8 mM final concentration), with the addition of 100 mg hydrated PVP (insoluble polyvinylpyrrolidone), centrifuged at 10,000 × g for 15 min, and then assayed with the Multimodel Plate Reader following the formation of trans-cinnamic acid from L-phenylalanine at 290 nm. One unit of PAL activity was defined as the amount of the enzyme that causes the increase in absorbance of 0.01 at 290 nm per h per milligram of protein. All tests were repeated at least three times.

### Biochemical analysis

Protein concentration was quantified using bovine albumin serum as a standard. Approximately 0.20 g of fresh leaves from each sample were transferred to a mortar and ground into powder in liquid nitrogen. For the extraction of soluble protein, the powder was extracted in 5 mL ddH_2_O for 30 min and was centrifuged at 3000 rpm for 10 min. Extract solutions (0.5 mL) were added to 2.5 mL coomassie brilliant blue G-25 and, after incubating for 5 min, measured with the Multimodel Plate Reader at 595 nm. For the extraction of soluble sugars, the powder was extracted in 10 mL 85% ethanol solution and incubated in a water bath at 60 °C for 20 min. Then, the extract solution was centrifuged at 3000 rpm for 10 min and the supernatant was collected. The pellet was re-extracted twice with the same solvent, and the supernatants were combined. The total supernatants were filtered through Whatman filter paper (Grade 1, Hangzhou Whatman-Xinhua filter paper Co., Ltd., Hangzhou, China). A filtrate of 0.1 mL was added to 5 mL anthrone-sulfuric acid and was assayed with the Multimodel Plate Reader at 620 nm. A standard curve was plotted with glucose.

### Observation and quantification of cotton leaf stomata

The second and third true leaves were cut off in pieces of 0.5 cm in size. The pieces were mounted on the microscope stage using conductive adhesive. The distributions of stomata were observed using a scanning electron microscope (TM-100, Hitachi, Japan) and were photographed to count the number and to measure the size of the stomata^43^. Each cotton line was planted in triplicate, and each repetition was observed 20 times randomly.

### Statistical analysis

Data are expressed as the means ± standard deviation (SD, n = 3). All statistical analyses were analyzed using SPSS software (SPSS Inc., version 13.0). Data were first normalized and then checked for normal distributions (Kolmogorov–Smirnov test) and homogeneity of variance (Levene's test). The normalizing transformation succeeded to improve the non-normality in the data. A two-way ANOVA with a repeated measures procedure of the generalized linear model (using 2 pairs of genotypes, “2 different genetic backgrounds”, and 2 pairs of transformations, “transgenic vs. non-transgenic treatments”, as independent factors, incubation time as a repeated measures factor) was applied for the following physiological characteristics: SOD, POD, PAL, protein and sugar^43^. The two-way ANOVA without repeated measures was used to determine the effects of transformation and genetic backgrounds on leaf stomatal density and sizes with no repeated measures. Differences were statistically significant at *P* < 0.05.

## Author Contributions

X.L. and B.L. conceived the research and designed the experiments. X.L., X.W. and C.D. wrote the main manuscript text. X.L. performed the experiments and data analysis. All authors discussed the results and commented on the manuscript.

## Figures and Tables

**Table 1:** Generalized Linear Mixed Model (GLMM) results for the overall effects on biochemical characteristics of transgenic cotton lines and conventional counterparts after inoculation with *F. oxysporum*. F- and *p*-values and associated degrees of freedom are listed

Effects	Degrees of freedom	F Value	*P* Value
SOD§			
Transgenic*	1:47	0.097	0.757
Genotype†	1:47	15.242	0.000
Pathogen infection‡	3:47	25.719	0.000
POD§			
Transgenic	1:47	0.238	0.628
Genotype	1:47	7.814	0.008
Pathogen infection	3:47	33.083	0.000
PAL§			
Transgenic	1:47	5.972	0.019
Genotype	1:47	34.969	0.000
Pathogen infection	3:47	29.865	0.000
Protein			
Transgenic	1:47	0.683	0.413
Genotype	1:47	18.277	0.000
Pathogen infection	3:47	235.372	0.000
Sugar			
Transgenic	1:47	2.100	0.155
Genotype	1:47	0.019	0.890
Pathogen infection	3:47	32.848	0.000

*“Transgenic” denotes transgenic lines vs. conventional counterparts.

†“Genotype” denotes 2 different paired genetic backgrounds.

‡“Pathogen infection” denotes 4 sampling times before and after pathogen infection.

§SOD is the abbreviation of Superoxide dismutase, POD is the abbreviation of Peroxidase, and PAL is the abbreviation of Phenylalanine ammonia lyase.

**Table 2:** Results of two-factorial analyses of variance on stomatal characteristics of transgenic cotton lines and conventional counterparts. F- and *p*-values and associated degrees of freedom are listed

Effects	Degrees of freedom	F Value	*P* Value
Longitudinal size of stomata			
Transgenic*	1:11	22.787	0.001
Genotype†	1:11	4.382	0.070
Transverse size of stomata			
Transgenic	1:11	8.901	0.018
Genotype	1:11	0.802	0.397
Stomatal density			
Transgenic	1:11	337.667	0.000
Genotype	1:11	6.873	0.031

*“Transgenic” denotes transgenic lines vs. conventional counterparts.

†“Genotype” denotes 2 different paired genetic backgrounds.

**Table 3:** The density and size of leaf stomata in transgenic cotton lines (Zhong-30, Zhong-41) and conventional counterparts (Zhong-16, Zhong-23)

		Sizes of leaf stomata (μm)	
Cotton lines	No. mm^−2^	Transverse	Longitudinal
Zhong-16	53.3 ± 4.48a	26.1 ± 2.11a	16.9 ± 1.51a
Zhong-30	36.7 ± 3.88b	28.6 ± 1.56b	18.0 ± 1.24b
Zhong-23	56.7 ± 6.16a	25.3 ± 1.72a	16.9 ± 1.06a
Zhong-41	27.4 ± 3.82b	27.4 ± 0.94b	17.5 ± 0.94b

Mean values and standard deviation of three replicates are presented. Significant differences in the variable means between transgenic lines and their counterparts are indicated by different letters (*P* < 0.05).
